# Multinucleated Giant Cancer Cells Produced in Response to Ionizing Radiation Retain Viability and Replicate Their Genome

**DOI:** 10.3390/ijms18020360

**Published:** 2017-02-08

**Authors:** Razmik Mirzayans, Bonnie Andrais, April Scott, Ying W. Wang, Piyush Kumar, David Murray

**Affiliations:** Department of Oncology, University of Alberta, Cross Cancer Institute, Edmonton, AB T6G 1Z2, Canada; bonnie.andrais@ahs.ca (B.A.); april.scott@ahs.ca (A.S.); ywwang@ualberta.ca (Y.W.W.); pkumar@ualberta.ca (P.K.); david.murray5@ahs.ca (D.M.)

**Keywords:** ionizing radiation, p53, p21^WAF1^, multinucleated giant cells, premature senescence, viability, proliferation, colony forming ability, 96-well plate XTT, single-cell MTT

## Abstract

Loss of wild-type p53 function is widely accepted to be permissive for the development of multinucleated giant cells. However, whether therapy-induced multinucleation is associated with cancer cell death or survival remains controversial. Herein, we demonstrate that exposure of p53-deficient or p21^WAF1^ (p21)-deficient solid tumor-derived cell lines to ionizing radiation (between 2 and 8 Gy) results in the development of multinucleated giant cells that remain adherent to the culture dish for long times post-irradiation. Somewhat surprisingly, single-cell observations revealed that virtually all multinucleated giant cells that remain adherent for the duration of the experiments (up to three weeks post-irradiation) retain viability and metabolize 3-(4,5-dimethylthiazol-2-yl)-2,5-diphenyl-tetrazolium bromide (MTT), and the majority (>60%) exhibit DNA synthesis. We further report that treatment of multinucleated giant cells with pharmacological activators of apoptosis (e.g., sodium salicylate) triggers their demise. Our observations reinforce the notion that radiation-induced multinucleation may reflect a survival mechanism for p53/p21-deficient cancer cells. With respect to evaluating radiosensitivity, our observations underscore the importance of single-cell experimental approaches (e.g., single-cell MTT) as the creation of viable multinucleated giant cells complicates the interpretation of the experimental data obtained by commonly-used multi-well plate colorimetric assays.

## 1. Introduction

Most human cell types that express wild-type p53 respond to moderate doses of ionizing radiation (e.g., 8 Gy) by exhibiting sustained nuclear accumulation of p21^WAF1^ (hereafter p21), which down regulates apoptosis and switches on the stress-induced premature senescence (SIPS) program [1,2,3,4]. SIPS is a growth-arrested state in which the cells acquire flattened and enlarged morphology, express the marker senescence-associated β-galactosidase (SA-β-gal), and cease to synthesize DNA, but remain viable and metabolically active. Radiation-induced SIPS is a prominent response of normal human fibroblasts [3,5] and p53 wild-type solid tumor-derived cell lines [1,2,3,4]. In addition, Li-Fraumeni syndrome fibroblasts [5] and some lung carcinoma cell lines [6] that lack wild-type p53 function also exhibit a high degree of SIPS upon exposure to ionizing radiation which appears to be mediated by p16^INK4A^ (p16).

The observation that ionizing radiation can trigger a sustained growth-arrested response was first reported by Puck and Marcus, who established the clonogenic survival method with cultured human cells, over half a century ago. In a series of landmark studies [7,8,9], these authors demonstrated that exposure of human cervical carcinoma (HeLa) cells to ionizing radiation results in the development of multinucleated giant cells (MNGCs) that grow very slowly, if at all, but nonetheless remain viable and secrete growth stimulating factors. This observation prompted the development of the feeder layer clonogenic assay, in which a “lawn” of heavily-irradiated feeder cells (which encompass MNGCs) is inoculated in a culture dish to promote the growth of test cells given graded doses of radiation [8]. The HeLa cell line used in those initial studies was subsequently shown to be infected with HPV 18, the E6 and E7 proteins of which disable the p53-p21 and the p16-pRB signaling pathways, respectively [10]. Not surprisingly, HeLa cells do not undergo SIPS after exposure to ionizing radiation [3].

The clonogenic survival assay developed by Puck and Marcus has been considered as the “gold standard” for evaluating radiosensitivity and chemosensitivity with different mammalian cell types. However, their intriguing observation that radiation exposure induces the formation of viable MNGCs has been largely overlooked. In fact, many authors equate multinucleation with cell death. Although a component of MNGCs that develop after therapeutic exposures is eliminated through apoptosis or other modes of cell death, compelling evidence reported in the past decade has demonstrated that the surviving MNGCs can contribute to cancer relapse by first entering a state of dormancy and ultimately giving rise to progeny with stem cell-like properties [11,12,13,14,15,16,17,18,19,20,21,22,23,24,25,26,27]. MNGCs can give rise to tumor-repopulating cells through different mechanisms, including nuclear budding or bursting similar to simple organisms like fungi [21,22,23,24,25,26]. The contribution of MNGCs to cancer recurrence following therapeutic exposures has been well documented for ovarian [21,24,25,26], breast [23] and colon cancers [22]. According to Weihua et al. [15], a single MNGC is sufficient to produce a metastatic tumor comprised mainly of mononuclear cells.

The growing complexity of cancer cell response to genotoxic stress, which involves not only early events (e.g., checkpoints and repair) but also late events such as SIPS and the creation of MNGCs, has been extensively reviewed by us and others (e.g., [18,20,28,29]). Despite this complexity, numerous short-term colorimetric assays are being increasingly used to assess radiosensitivity and chemosensitivity [30,31,32,33,34,35]. Such assays are often performed in a multi-well plate format and the results (absorbance or fluorescence) are recorded in a plate reader. Although all multi-well plate colorimetric assays provide a read out of combined effects associated with transient cell cycle checkpoints (pro-survival mechanisms), growth inhibition (which can be reversible under some conditions) and cell death, the change of absorbance/fluorescence in genotoxin-treated wells relative to control (solvent-treated) wells is often assumed to reflect loss of viability and hence lethality. This assumption would be reasonable only if the number and size of cells in treated and control wells would be identical at the time of reading the plates; however, this is rarely, if ever, the case.

The goal of the studies reported here was to determine whether the development of viable MNGCs after exposure to ionizing radiation is a prominent response of human solid tumor-derived cell lines that lack p21 or wild-type p53 functions, and if so, how this response might impact on the interpretation of data obtained with cell-based assays in general, and short-term colorimetric assays in particular.

## 2. Results

Human cells lacking p21 or wild-type p53 function escape cell cycle checkpoints after exposure to high doses of ionizing radiation (≥10 Gy) and execute mitosis despite carrying high levels of DNA radioproducts and chromosomal aberrations, leading to the creation of MNGCs [3,36]. In addition to multinucleation, radiation exposure can trigger the creation of mono-nucleated cells with massive DNA content [20,36]. Mono-nucleated giants can develop through different routes, including endoreduplication and nuclear fusion of multinucleated cells. For simplicity, however, herein we will use “MNGCs” to reflect both multinucleated giant cells and mono-nucleated giant cells with highly enlarged nuclei. It is important to note that such mono-nucleated giant cells differ from senescent cells that exhibit enlarged and flattened cellular morphology, but exhibit a diploid DNA content [5,37].

We determined whether the development of MNGCs is a frequent response of p53/p21-deficient cells following exposure to moderate doses of ionizing radiation (between 2 and 8 Gy) that are typically used in the colony formation assay. The first series of experiments was performed with p21 knockout (HCT116p21−/−) and p53 knockout (HCT116p53−/−) derivatives of the HCT116 colon carcinoma cell line (Section 2.1, Section 2.2, Section 2.3 and Section 2.4). Next, we evaluated other cancer cell lines, including breast cancer cell lines that express mutant p53 (Section 2.5). In addition, we determined the contribution of MNGCs to radiosensitivity as measured by short-term and long-term assays (Section 2.6).

### 2.1. Impact of p21 or p53 Loss on the Formation of MNGCs in HCT116 Cells Exposed to Ionizing Radiation

**Figure 1:**
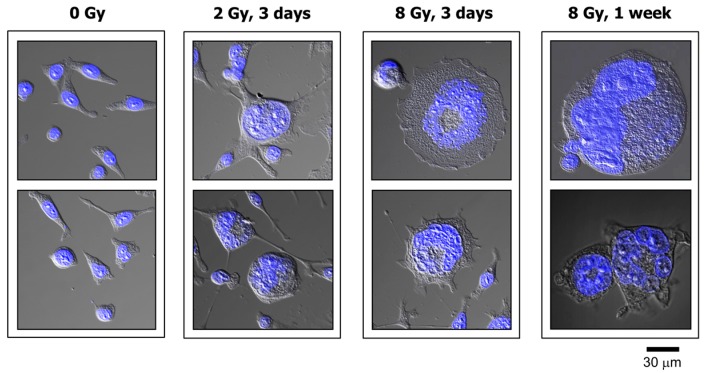
Confocal microscopy images showing nuclear morphology of HCT116p21−/− cells before and at indicated times after irradiation. Nuclear content (DAPI staining) is shown in blue. All images were acquired at the same magnification.

**Figure 2:**
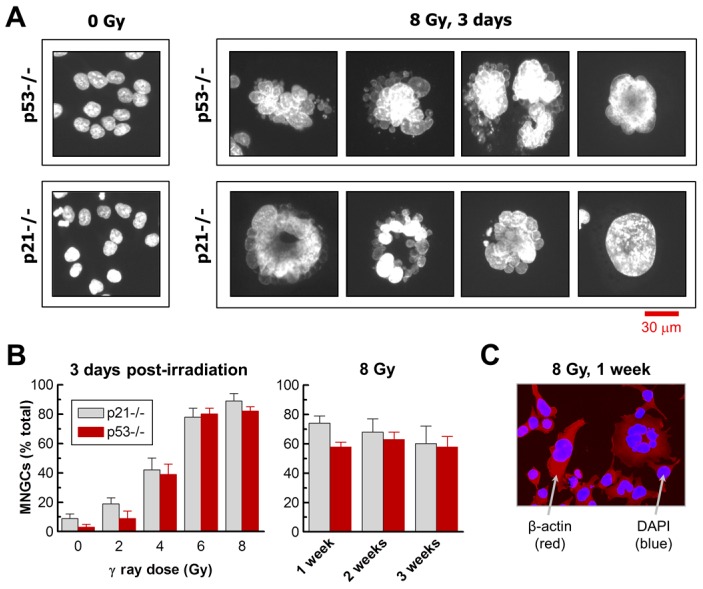
(**A**) Fluorescence images showing the nuclear morphology of HCT116p53−/− and HCT116p21−/− cells before and after irradiation. Nuclear content (DAPI) is shown in white. All images were acquired at the same magnification; (**B**) Proportion of MNGCs arising in cultures of the indicated cell lines following radiation exposure; Bars, SE (Standard error). At ~1 week post-irradiation, some dishes contained regions with colonies consisting of heterogeneous cells. The image of a small colony is shown in (**C**); Cells in such colonies were not used for the data presented in panel (**B**).

Exposure of HCT116p21−/− and HCT116p53−/− cultures to radiation followed by incubation for 3 days resulted in the development of MNGCs in a dose-dependent manner (Figure 1 and Figure 2). In both cultures, the proportion of MNGCs increased from ~10% to ~80% after exposure to 2 Gy and 8 Gy, respectively (Figure 2B). Not only did a high proportion of MNGCs remain adherent to the culture dish for the duration of the experiments (up to three weeks after irradiation), but the nuclear content of most of them increased with increasing post-irradiation incubation time (Figure 1).

Exposure of these cultures to radiation followed by incubation for long times (>3 days) also resulted in the appearance of floating cells, presumably reflecting late apoptosis and other modes of cell death. The purpose of the current work was not to study the basis and fate of floating cells. However, in some experiments we did evaluate the morphology of floating cells that appeared post-irradiation (8 Gy) and observed only a fraction (<1%) to contain a highly enlarged nucleus or multiple nuclei (data not shown).

**Figure 3:**
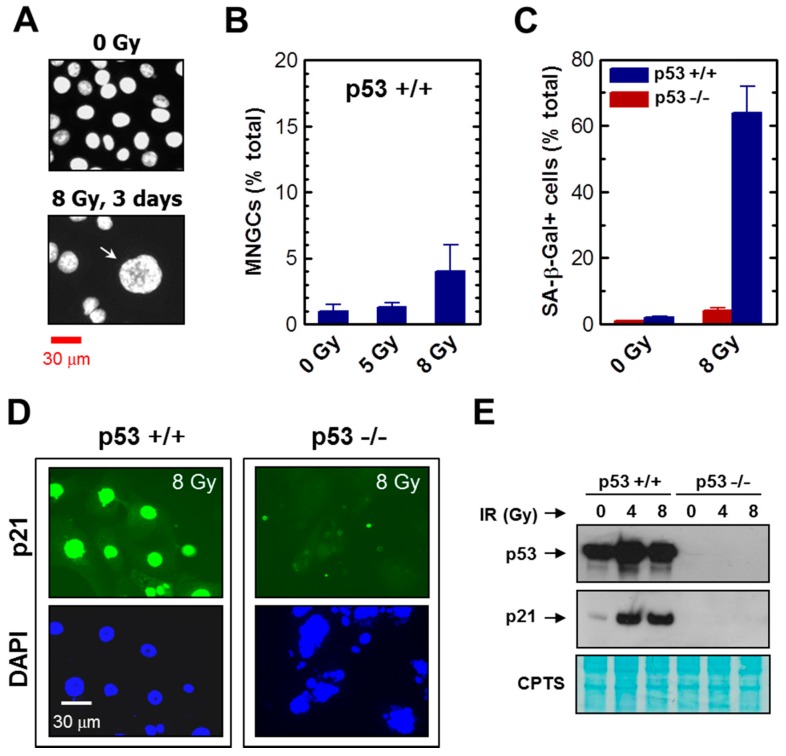
(**A**) Fluorescence images showing the nuclear morphology of HCT116 cells before and after irradiation. Nuclear content (DAPI) is shown in white. The two images were acquired at the same magnification. The arrow shows a mononucleated giant cell; (**B**) Proportion of MNGCs arising in HCT116 cultures following radiation exposure. Bars, SE; (**C**) Percentages of cells exhibiting SA-β-gal expression in HCT116 parental (p53+/+) and HCT116p53−/− cultures before and seven days after irradiation. Bars, SE; (**D**) Representative images showing p21 levels in the indicated cultures seven days after irradiation; (**E**) Western blot analysis of global p53 and p21 protein levels in the indicated cultures before and six days after irradiation. CPTS staining of the membrane (lower image) confirmed equal loading. IR, ionizing radiation.

We also evaluated the parental HCT116 cultures for the development of MNGCs post-irradiation. Exposure to high doses of radiation followed by incubation for three days did result in the development of MNGCs in HCT116 cultures, albeit at a very low frequency (<5% after 8 Gy irradiation) (Figure 3A,B). On the other hand, radiation exposure of HCT116 cultures (but not HCT116p53−/− cultures) triggered SIPS in a high proportion of cells (>60%), as determined by induction of p21 and positive staining in the SA-β-gal assay (Figure 3C–E). These observations are consistent with results reported by Lindgren et al. [37] demonstrating that radiation exposure (6 Gy) induces a high degree of SIPS (but not polyploidy) in HCT116 cultures, and a high degree of polyploidy (but not SIPS) in HCT116p53−/− cultures. 

### 2.2. MNGCs Retain Viability

**Figure 4:**
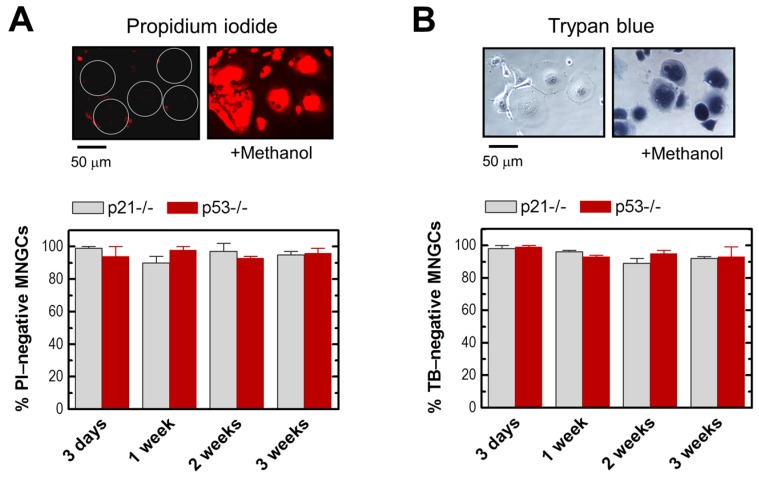
(**A**) Viability of MNGCs created in HCT116p53−/− cultures at seven days after radiation exposure (8 Gy), evaluated by the PI-exclusion assay. The two images were taken from the same field, before (left image) and after (right image) addition of methanol to the culture medium to permeabilize the cells. Circles mark viable giant cells. The lower panel shows quantitative data for HCT116p53−/− and HCT116p21−/− cultures at different times post-irradiation. Bars, SE; (**B**) Viability of the same cultures evaluated by the TB-exclusion assay.

Viability of MNGCs was determined by both propidium iodide (PI) exclusion and trypan blue (TB) exclusion assays. Our approach [5,38] does not involve cell detachment (e.g., by exposure to trypsin) to avoid creating false positives due to transiently damaging the cell membrane. For the PI exclusion assay, cells were exposed to radiation (8 Gy) and incubated for different times between three days and three weeks. PI (10 μg/mL) was added to the culture medium and the cells were examined under a fluorescence microscope. For the TB exclusion assay, after irradiation and post-irradiation incubation as above, the medium was removed and the cells were overlaid with PBS containing TB (0.2%) and examined under a bright field microscope. The results obtained with the HCT116p21−/− and HCT116p53−/− cell lines are presented in Figure 4. Virtually all MNGCs that remained adherent at any given time post-irradiation excluded PI and TB, and are thus considered to be viable.

**Figure 5:**
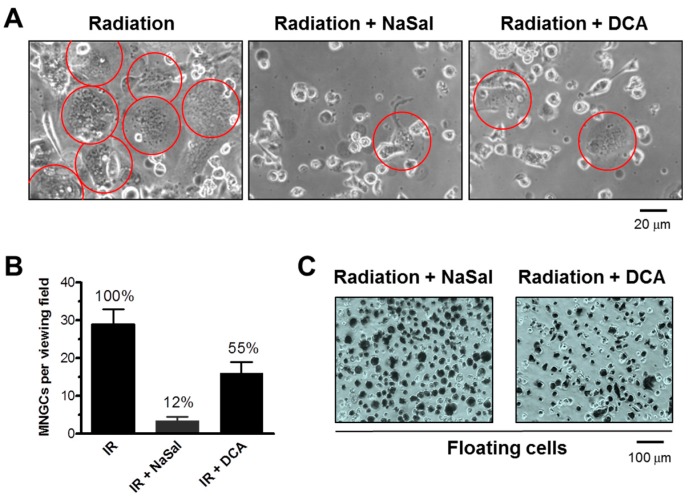
(**A**) Phase-contrast photomicrographs showing the effect of NaSal and DCA on the MNGCs that developed in HCT116p53−/− cultures following irradiation (4 Gy). Circles mark MNGCs that remained adherent to the culture dish under these conditions; (**B**) Proportion of adherent MNGCs after the indicated treatments. The data are expressed as numbers of MNGCs per viewing field for each treatment condition, as well as the percent values for radiation (IR) plus drug-treated samples relative to IR only samples (set at 100%). Bars, SE; (**C**) Bright-field microscopy images depicting loss of viability of floating cells, evaluated by the TB-exclusion assay.

As a positive control, we treated MNGCs with pharmacological stimulators of apoptosis to trigger their demise. We tested the effects of sodium salicylate (NaSal), an inhibitor of the p38 MAPK (mitogen-activated protein kinase) [39], and dichloroacetate (DCA), a modulator of glucose metabolism [40]. In these experiments, cells were plated in 60-mm dishes (10^5^ cells/dish), exposed to radiation (4 Gy), and incubated for five days to allow the development of MNGCs. The medium was then replaced with medium containing NaSal (5 mg/mL), DCA (8 mg/mL), or solvent. The cells were incubated for 24 h and the frequency of adherent MNGCs was determined under a phase-contrast microscope. Next, the floating cells were evaluated for viability by the TB-exclusion assay. The results obtained with HCT116p53−/− cultures are presented in Figure 5. NaSal and DCA caused cell detachment of ~90% and ~45% of MNGCs, respectively, and the majority of floating cells were TB positive. Similar results were obtained with HCT116p21−/− cultures (data not shown).

### 2.3. MNGCs Exhibit Metabolic Activity

As mentioned earlier, short-term colorimetric assays are widely used for evaluating the radiosensitivity of different mammalian cell types. Such assays are typically performed between 24 and 72 h post-irradiation. One of the most commonly-used colorimetric assays is based on the ability of viable cells to convert the yellow 3-(4,5-dimethylthiazol-2-yl)-2,5-diphenyl-tetrazolium bromide (MTT) dye to its purple formazan metabolite. The MTT formazan metabolite is water insoluble, requiring a solvent-treatment step (e.g., with DMSO) to dissolve the metabolite and facilitate its evaluation by a plate reader [41]. While this property is considered a pitfall for multi-well approaches, it can actually be exploited for microscopic assessment of the metabolic activity of individual cells in the absence of solvent treatment. MTT reduction in living cells can be visualized as intracellular formazan granules shortly (<30 min) after adding MTT to the culture medium [42]. This is followed by the appearance of needle-like formazan crystals which are exocytosed, resulting in a hair-like morphology on the cell surface.

**Figure 6:**
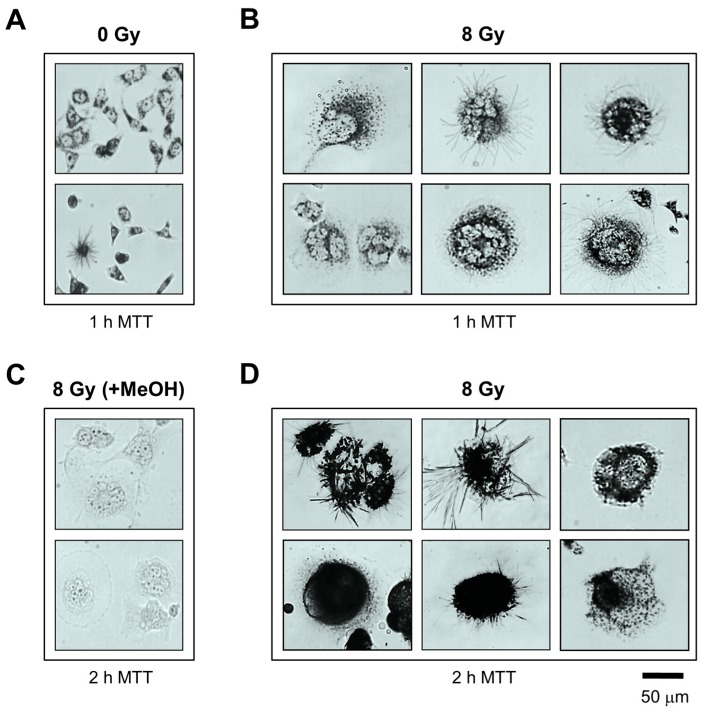
Bright-field microscopy images depicting the metabolic activity of HCT116p53−/− cultures: before (**A**); and after (**B**–**D**) 8-Gy irradiation. Metabolic activity was measured by the ability of the cells to convert the yellow MTT to its purple formazan metabolite, appearing as dark granules and crystals; As a negative control, cells in some dishes were first treated with methanol to inhibit their metabolic activity, and then incubated with MTT (**C**). All images were acquired at the same magnification.

**Figure 7:**
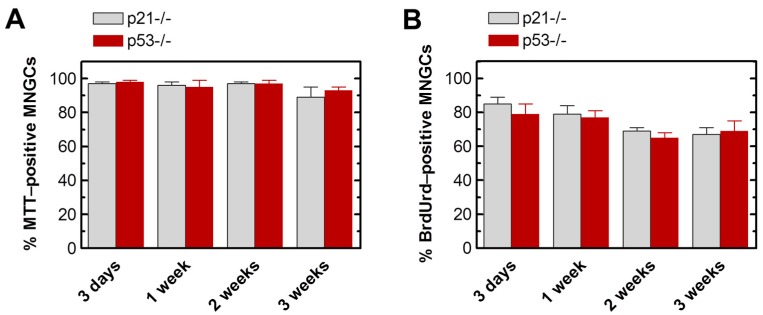
Percentages of: MTT-positive (**A**); and BrdUrd-positive (**B**) MNGCs in HCT116p21−/− and HCT116p53−/− cultures at indicated times after irradiation (8 Gy). Bars, SE.

We used the single-cell MTT assay to assess the metabolic activity of MNGCs. To this end, cultures of HCT116p21−/− and HCT116p53−/− cell lines were exposed to radiation and incubated for different times as for the vital dye exclusion assays. At the time of metabolic activity assessment, the culture medium was replaced with fresh medium containing MTT and the cells were returned to the incubator as recommended by the supplier for the standard multi-well method. Bright-field microscopy images were acquired in 15-min intervals, after which the cells were returned to the incubator. Incubation of MNGCs with MTT led to the formation of formazan granules in ~15 min, followed by massive amounts of formazan formation by ~1 h such that MNGCs turned purple, followed by the appearance of hair-like morphology. As shown in Figure 6, at 1 (Figure 6B) and 2 h (Figure 6D) after incubation with MTT, large amounts of formazan granules and crystals were present in virtually all MNGCs, indicating that they retained metabolic activity. In Figure 7A, it can be seen that these MTT-positive MNGCs persisted in cultures of both cell lines for up to three weeks after irradiation.

### 2.4. MNGCs Exhibit DNA Synthesis

**Figure 8:**
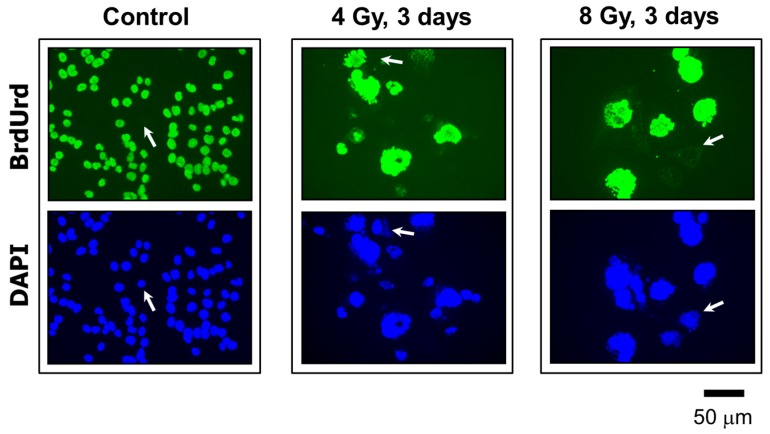
Representative images showing proliferating cells (BrdUrd-positive; green) and total cells (DAPI; blue) in HCT116p53−/− cultures acquired before and three days after irradiation. In all experiments, BrdUrd was added to the culture medium for the final 24 h of the incubation period to allow its incorporation into genomic DNA. Arrows show some cells that did not incorporate BrdUrd under these conditions. All images were acquired at the same magnification.

The ability of MNGCs to replicate their genome was assessed by the bromodeoxyuridine (BrdUrd) immunofluorescence assay [5]. This thymidine analog incorporates into DNA during replication, which can be detected by the use of a BrdUrd-specific antibody and standard “DNA” fluorescence techniques. The results are presented in Figure 7B and Figure 8. In these experiments, cultures were exposed to radiation (4 or 8 Gy) or sham-exposed, incubated for different times (between three days and three weeks) in growth medium (without BrdUrd); BrdUrd was added to the culture medium for the final 24 h incubation period to allow its selective incorporation into the genome of replicating cells. Under these conditions, the majority of MNGCs that were formed in irradiated HCT116p21−/− and HCT116p53−/− cultures exhibited DNA synthesis as judged by BrdUrd incorporation.

### 2.5. Development of Viable MNGCs in Mutant p53-Expressing Cancer Cell Lines Exposed to Ionizing Radiation

**Figure 9:**
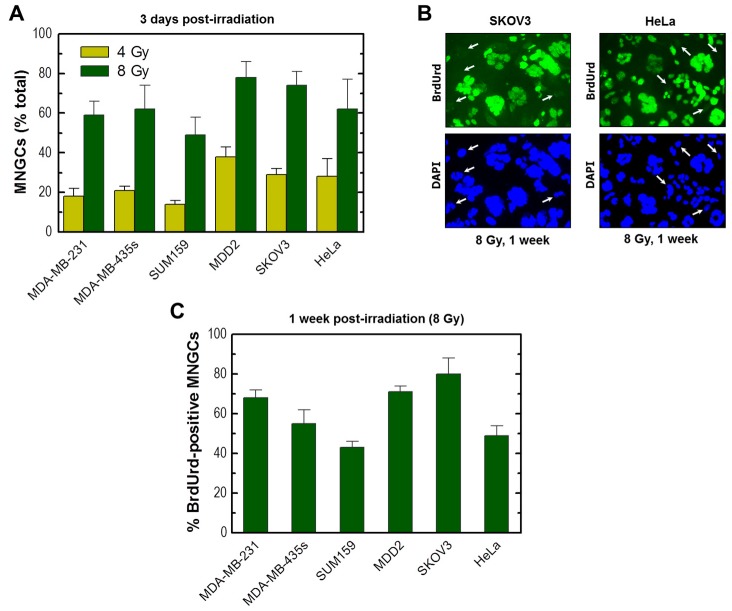
(**A**) Development of MNGCs in the indicated cancer cell lines 72 h after exposure to ionizing radiation. Bars, SE; (**B**) BrdUrd immunostaining of the indicated cultures that were exposed to radiation (8 Gy) and incubated for six days in fresh medium and for a further 24 h in medium containing BrdUrd. Images were acquired for regions in the dish that contained aggregates of cells (mini-colonies). Arrows show some cells that did not incorporate BrdUrd under these conditions; (**C**) Percentages of BrdUrd-positive MNGCs post-irradiation in the indicated cell lines. Bars, SE.

These studies were extended to the breast cancer cell lines MDA-MB-231, MDA-MB-435s, SUM159 and MDD2 that express mutant p53 [36,37,38]. The mutant p53 in MDA-MB-231 cells is auto-phosphorylated (e.g., on Ser15) and exhibits gain-of-function properties [43,44]. We also examined the HeLa cervical carcinoma and SKOV3 ovarian carcinoma cell lines that are p53-deficient due to HPV 18 infection [10] and truncating *TP53* mutation [45], respectively. Radiation exposure followed by incubation for three days (Figure 9A) or longer times (data not shown) led to the development of MNGCs in cultures of all cell lines; the majority (>90%) of these MNGCs retained viability and a high proportion (>40%) exhibited DNA synthesis (Figure 9B,C, and data not shown).

### 2.6. Impact of MNGCs on Radiosensitivity Measured by Growth Inhibition, Colony Formation and 96-Well Plate (XTT) Assays

**Figure 10:**
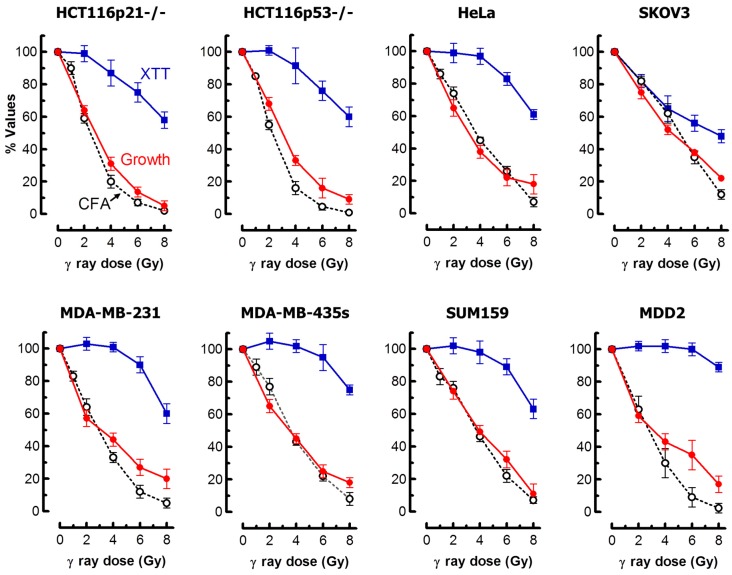
Radiosensitivity of the indicated cell lines evaluated by growth inhibition and XTT assays, both performed three days after irradiation. The results obtained by the colony formation assay are also shown. Bars, SE; CFA, colony forming ability.

These results suggest that MNGCs can contribute to growth inhibition and loss of clonogenic potential after irradiation, but might complicate the interpretation of the radiosensitivity data obtained with multi-well colorimetric assays. Specifically, in the latter assays, the creation of viable and metabolically active MNGCs would be expected to skew the radiation dose-response curve towards radioresistance. We performed a comprehensive radiosensitivity study with our panel of cell lines and confirmed these predictions. The results are presented in Figure 10.

For the 96-well plate assay, we used XTT (2,3-bis-(2-methoxy-4-nitro-5-sulfophenyl)-2*H*-tetrazolium-5-carboxanilide) rather than MTT because XTT yields a water soluble formazan and thus there is no need for an additional solubilization step. In eight out of the nine cell lines that we examined, there was a remarkable discordance in the degree of radiosensitivity measured by XTT compared to growth inhibition and colony formation assays. In HeLa cells, for example, exposure to a 4-Gy dose of radiation caused little effect in the XTT assay (~100% “survival”) but resulted in significant growth inhibition and loss of colony-forming ability (~40% “survival”).

In SKOV3 cells, however, the XTT, growth inhibition and clonogenic assays yielded comparable results. The basis for such different outcomes for different cell lines determined by the XTT assay under identical experimental conditions remains unknown. We considered the possibility that the cell number used for some cell lines might not have been optimal. However, this does not appear to be likely because using lower cell numbers for selected cell lines (e.g., HCT116p53−/−) did not impact on the degree of radiosensitivity as measured by this assay (data not shown).

An important take-home message of the results presented in Figure 10 is that the conventional growth inhibition assays used by us [46] and others [47], which can be completed within five days from seeding the cells, generate radiosensitivity results comparable to those obtained with the laborious and time-consuming clonogenic assays. In addition to reproducibility and relative ease of performance, the growth inhibition assay enables microscopic examination to predict the long-term fate of adherent cells. To this end, cells in one set of dishes can be fixed, either at the time of cell counting post-irradiation or upon further incubation, and evaluated for different responses (e.g., apoptosis, SIPS and multinucleation) using a battery of single-cell methods.

## 3. Discussion

The Nomenclature Committee on Cell Death (NCCD) has made recommendations for the classification of cell death and molecular definitions of cell death subroutines [48]. Specifically, the NCCD has recommended that a cell should be considered dead when the integrity of its plasma membrane is lost, as defined by the incorporation of vital dyes such as PI, and/or when the cell, including its nucleus, has undergone complete fragmentation into apoptotic bodies. Recently, we reviewed the current knowledge on radiation-induced responses that can lead to lethality and, paradoxically, can promote tumor repopulation depending on context [29]. These include activation of caspases (e.g., caspase 3) and growth arrest through SIPS and/or multinucleation.

In the current study, we have demonstrated that human solid tumor-derived cell lines lacking p21 or wild-type p53 activity respond to moderate (“survival-curve-range”) doses of ionizing radiation by exhibiting growth arrest that is attributed to the creation of MNGCs. In addition, the adherent MNGCs retain membrane integrity and metabolize MTT, and the majority exhibit DNA synthesis, when evaluated at later times (between three days and three weeks) post-irradiation. These observations, in concert with the growing body of evidence connecting therapy-induced multinucleation to disease relapse [12,13,14,15,16,17,18,19,20,21,22,23,24,25,26,27], suggest that identifying pharmacological/biological agents capable of inducing the demise of MNGCs before they give rise to tumor-repopulating progeny may have important clinical ramifications. It is intriguing in this regard that Puck and Marcus noted six decades ago that the application of Newcastle disease virus to irradiated HeLa cultures led to the destruction of MNGCs [7].

Although multi-well colorimetric assays (e.g., MTT, XTT, and CellTiter-Blue) are widely used to evaluate radiosensitivity, the development of viable MNGCs following irradiation complicates the interpretation of the optical density recordings. Not surprisingly, in the current study, the 96-well plate XTT assay markedly underestimated radiosensitivity when compared to growth inhibition and colony formation assays. Thus, for studies that do not require high throughput screening, the single-cell MTT assay appears to be a more reliable tool to determine cell viability/metabolic activity as compared to optical density measurements performed in a multi-well format.

In cell-based assays that are commonly used to identify novel cancer therapeutic approaches, determining a 50% effect—so-called IC_50_ (inhibiting concentration, 50%) for chemical compounds and ID_50_ (inhibiting dose, 50%) for radiation—is considered an informative indicator of genotoxicity of clinical relevance. In the current study, the ID_50_ values measured by the growth inhibition and colony formation assays were similar in all nine cell lines that we examined. The growth inhibition assay circumvents some of the difficulties associated with the clonogenic assay, such as poor quality of the single-cell preparations and/or poor cloning efficiencies in certain cell lines [43]. In addition, the growth inhibition assay is completed within five days from the seeding of the cells.

Irrespective of the type of assay employed, the take-home message of a large body of data generated in recent decades, including the results presented here, is not different from what Puck and Marcus reported for the HeLa cervical carcinoma cell line sixty years ago [7]. Namely, genotoxic treatment predominantly triggers cancer cell dormancy rather than lethality.

Our current observation that radiation exposure triggers the development of MNGCs in solid-tumor-derived cell lines exhibiting aberrant p53/p21 function is not surprising. However, the frequency of such events following moderate dose irradiation and the finding that a large proportion of MNGCs remain viable, metabolize MTT and exhibit DNA synthesis is potentially alarming, given that a single multinucleated cancer cell has been reported to be sufficient to promote metastatic tumors in a mouse model [15]. It will be important to determine the impact on creation of MNGCs of multiple 2-Gy doses to mimic the scenario with conventional fractionated radiotherapy. We anticipate that daily fractionated irradiation will result in a marked enrichment of MNGCs given that such cells are highly resistant to genotoxic stress compared to mononucleated (diploid/near diploid) cells [15].

Further research will hopefully lead to identifying a molecular basis for the long-term survival of MNGCs which will set the stage for novel therapeutic approaches to simultaneously control the initial tumor growth and prevent disease recurrence.

## 4. Materials and Methods

### 4.1. Cells and Culture Conditions

The human colon carcinoma cell line HCT116 and its p21 or p53 homozygous knockout derivatives were generous gifts of Bert Vogelstein (Johns Hopkins University, Baltimore, MD, USA). All other cancer cell lines used in the present study were purchased from the American Type Culture Collection (Rockville, MD, USA). Cells were cultured as monolayers in DMEM/F12 nutrient medium supplemented with 5% (*v*/*v*) fetal bovine serum, 1 mM L-glutamine, 100 IU/mL penicillin G and 100 µg/mL streptomycin sulfate in a 37 °C chamber incubator providing a humidified atmosphere of 5% CO_2_ in air. All cultures were free of *Mycoplasma* contamination. 

### 4.2. Reagents

Mouse monoclonal antibodies to p21 (sc-187), p53 (DO-1) and β-actin (C4) were purchased from Santa Cruz Biotechnology (Santa Cruz, CA, USA). A mouse monoclonal antibody to BrdUrd (clone BU-33) was purchased from Sigma (St. Louis, MO, USA). An Alexa Fluor 488 secondary antibody (goat anti-mouse IgG) was purchased from Invitrogen (Eugene, OR, USA). The fluorescent tracers 4′, 6-diamidino-2-phenylindole (DAPI) (Invitrogen), Hoechst 33258 (Invitrogen), propidium iodide (PI) (Sigma), the vital dye trypan blue (TB) (Sigma), and the tetrazolium dyes 3-(4,5-dimethylthiazol-2-yl)-2,5-diphenyl-tetrazolium bromide (MTT) and 2,3-bis-(2-methoxy-4-nitro-5-sulfophenyl)-2*H*-tetrazolium-5-carboxanilide salt (XTT) (Roche Diagnostics, Penzberg, Germany) were used as recommended by the manufacturers. Dichloroacetate (DCA) and sodium salicylate (NaSal) (both from Sigma) were dissolved in distilled water and stored at 4 °C.

### 4.3. Radiation Exposure

Exposure to ^60^Co γ-rays was performed in a Gammacell 220 unit as described [49].

### 4.4. Immunoblot and Immunofluorescence Techniques

Global levels of specific proteins were evaluated by immunoblotting as described [50]. Protein immunofluorescence and BrdUrd immunofluorescence assays were performed as described [51].

### 4.5. SA-β-Gal Assay

SA-β-gal staining was performed using the kit supplied by Cell Signaling Technology (Beverly, MA, USA) as described [51].

### 4.6. Single-Cell MTT Assay

Cell metabolic activity was determined by the single-cell MTT assay using conditions recommended by the supplier (Roche Diagnostics, Penzberg, Germany) for the standard multi-well plate cell proliferation method. To this end, cells were plated in 35-mm dishes (~20,000 cells/2 mL medium/dish), exposed to radiation (or sham-irradiated) and incubated for different times between 3 days and 3 weeks, with weekly medium renewal as needed. At the time of metabolic assessment, the culture medium was replaced with fresh medium containing MTT (final concentration, 0.5 mg/mL) and the cells were returned to the incubator. Bright-field microscopy images were acquired in 15-min intervals for up to 2 h after incubation with MTT.

### 4.7. XTT Cell Proliferation Assay

The XTT cell proliferation assay was performed according to the instructions provided with the kit (Roche Diagnostics) with minor modifications. Briefly, cells were plated in 100-mm tissue culture dishes and incubated until 90% confluency. The cells were detached by the use of trypsin and suspended in culture medium at a density of 10,000 cells/mL. Cell suspensions were exposed to doses of ionizing radiation between 2 and 8 Gy; sham-irradiated cells served as controls. Cells were plated in 96-well tissue culture plates at a density of 2,000 cells per well in 200 µL of medium and incubated for 48 h. XTT labelling reagent and electron-coupling reagent were then added according to the instructions provided with the kit, followed by incubation for 18 h. Absorbance of the wells was determined at 492-nm using a plate reader (Fluostar Optima FL, BMG Labtech, Ortenberg, Germany).

### 4.8. Colony Formation Assay

Cells were seeded in 60-mm dishes (~300 cells/5 mL medium/dish) and exposed to doses of ionizing radiation between 1 and 8 Gy (or sham-irradiated). The time interval between cell seeding and irradiation was <1 h. Cells were incubated for 10 days without medium renewal and evaluated for colony-forming ability as described [38].

### 4.9. Growth Inhibition Assay

Cells were plated in 60-mm dishes (10^5^ cells/5 mL medium/dish), incubated overnight, and then exposed to various doses of ionizing radiation between 2 and 8 Gy (or sham-irradiated). After incubation for 3 days, adherent cells were harvested by the use of trypsin and counted by a cell counter (Coulter, Hialeah, FL, USA). The cell inoculum size (number of adherent cells per dish at the time of irradiation) was also determined; this number was subtracted from the number of cells per dish measured 3 days after irradiation (or sham-irradiation). Growth inhibition curves were generated by plotting the extent of cell growth in irradiated dishes (expressed as a percentage of cells in sham-irradiated dishes) as a function of radiation dose.

## Abbreviations

MTT	3-(4,5-Dimethylthiazol-2-yl)-2,5-diphenyl-tetrazolium bromide
XTT	2,3-Bis-(2-Methoxy-4-nitro-5-sulfophenyl)-2*H*-tetrazolium-5-carboxanilide
SA-β-gal	Senescence-associated β-galactosidase
SIPS	Stress-induced premature senescence
TB	Trypan blue
PI	Propidium iodide
CPTS	Copper(II) phthalocyanine-tetrasulfonic acid tetrasodium salt
PBS	Phosphate-buffered saline
DCA	Dichloroacetate
NaSal	Sodium salicylate
DMSO	Dimethyl sulfoxide
BrdUrd	Bromodeoxyuridine
HPV	Human papillomavirus
ID_50_	Inhibiting dose, 50%
IC_50_	Inhibiting concentration, 50%
DAPI	4′,6-diamidino-2-phenylindole
CFA	Colony forming ability
SE	Standard error
